# P-2136. Bloodstream Infections in Hematologic Malignancies: Incidence Across Clinical Scenarios

**DOI:** 10.1093/ofid/ofaf695.2300

**Published:** 2026-01-11

**Authors:** Jessica Seidelman, Patrick C Tam, Julia A Messina, Alicia Gray, JoAnn Liu, Kimberiey Milliam, Jonathan Huggins, Jennifer Saullo

**Affiliations:** Duke University School of Medicine, Durham, NC; Duke University School of Medicine, Durham, NC; Duke University, Apex, North Carolina; Duke Univrsity, Durham, North Carolina; Duke University, Apex, North Carolina; Duke University, Apex, North Carolina; Duke University Hospital, Durham, NC; Duke University, Apex, North Carolina

## Abstract

**Background:**

Blood culture (BCx) diagnostic stewardship is key to maximizing diagnostic utility while reducing inefficient use of health resources without compromising patient outcomes. Current BCx algorithms rely on bloodstream infection (BSI) incidence to guide clinical decision-making, yet most studies informing these protocols are limited to immunocompetent populations. Given the unique risks faced by patients with hematologic malignancies, we sought to determine the incidence of BSI across various clinical indications in this cohort.

**Methods:**

We performed a retrospective chart review at an academic medical center from 10/2023-2/2024, analyzing all consecutive BCxs obtained in the hematologic malignancy inpatient and outpatient units. Using a standardized adjudication process, we categorized BCx results as negative, true positive, or contaminant. The primary objective was to assess BSI incidence across different clinical scenarios.

**Results:**

1,511 BCxs were collected during the study period. The most common diagnoses in patients who had BCx sampling included acute myeloid leukemia (518, 34.3%), non-Hodgkin’s lymphoma (259, 17.1%), and multiple myeloma (245, 16.2%). The majority BCxs were performed inpatient (1368, 90.5%). Among these, 1,479 (97.9%) were standard anaerobic/aerobic cultures, 32 (1.1%) were fungal/mycobacterial cultures. The most common indications for BCxs were neutropenic fever (655, 43.3%), non-neutropenic fever (180, 11.9%), and assessment for clearance of bloodstream infection (123, 8.1%). Overall, 1,373 (90.9%) cultures were negative, 121 (8.0%) were true positives, and 17 (1.8%) were contaminants. The highest true positive rates were observed for skin/soft tissue or bone infections (10, 18.2%), assessment for clearance of bacteremia (20, 16.3%), and non-neutropenic fever (21, 11.7%). None of the 32 fungal/mycobacterial cultures yielded positive results.

**Conclusion:**

Our study provides an updated assessment of BSI incidence in patients with hematologic malignancies, highlighting key clinical scenarios where BCxs are most likely to yield true positives. These findings can inform future efforts to refine BCx diagnostic stewardship in this high-risk population, ensuring that testing strategies are both effective and judicious.

**Disclosures:**

Jessica Seidelman, MD, MPH, 3M: Expert Testimony|Nichols & Associates: Expert Testimony|osteal: Advisor/Consultant|UptoDate: Content Contributor Julia A. Messina, MD, MHS MS, Seres: Advisor/Consultant|UpToDate: Royalties Jennifer Saullo, MD, Pharm D, RMEI Medical Education: Honoraria|UpToDate: Royalties

